# Mucoid *Acinetobacter baumannii* enhances anti-phagocytosis through reducing C3b deposition

**DOI:** 10.3389/fmed.2022.879361

**Published:** 2022-09-15

**Authors:** Xiaoxia Gong, Qian Zhao, Yifan Wu, Hongwei Zhou, Shuangyang Ding, Kui Zhu

**Affiliations:** ^1^National Center for Veterinary Drug Safety Evaluation, College of Veterinary Medicine, China Agricultural University, Beijing, China; ^2^Guangdong Laboratory for Lingnan Modern Agriculture, Guangzhou, China; ^3^Second Affiliated Hospital, School of Medicine, Zhejiang University, Hangzhou, China

**Keywords:** *A. baumannii*, anti-phagocytosis, C3b deposition, capsule, mucoidity

## Abstract

**Background:**

Multidrug resistant (MDR) *Acinetobacter baumannii* causes serious infections in intensive care units and is hard to be eradicated by antibiotics. Many *A. baumannii* isolates are identified as the mucoid type recently, but the biological characteristics of mucoid *A. baumannii* and their interactions with host cells remains unclear.

**Methods:**

The mucoid phenotype, antimicrobial susceptibility, biofilm-forming ability, acid resistance ability, peroxide tolerance, and *in vivo* toxicity of clinical ICUs derived *A. baumannii* isolates were first investigated. Secondly, the phagocytic resistance and invasive capacity of *A. baumannii* isolates to macrophages (MH-S, RAW264.7) and epithelial cells (A549) were analyzed. Furthermore, the abundance of C3b (complement factor C3 degradation product) deposition on the surface of *A. baumannii* was investigated. Last, the relationship between C3b deposition and the abundance of capsule in *A. baumannii* isolates were analyzed.

**Results:**

These *A. baumannii* strains showed different mucoid phenotypes including hyper mucoid (HM), medium mucoid (MM), and low mucoid (LM). All tested strains were MDR with high tolerance to either acid or hydrogen peroxide exposure. Notably, these mucoid strains showed the increase of mortality in the *Galleria mellonella* infection models. Besides, the HM strain exhibited less biofilm abundance, higher molecular weight (MW) of capsule, and greater anti-phagocytic activity to macrophages than the LM strain. Together with the increased abundance of capsule, high expression of *tuf* gene (associated with the hydrolysis of C3b), the HM strain effectively inhibits C3b deposition on bacterial surface, resulting in the low-opsonization phenotype.

**Conclusion:**

Capsular characteristics facilitate the anti-phagocytic activity in hyper mucoid *A. baumannii* through the reduction of C3b deposition. Mucoid *A. baumannii* exhibits high phagocytosis resistance to both macrophages and epithelial cells.

## Introduction

The increasing prevalence of pan drug-resistant Gram-negative bacteria, especially the carbapenem resistant *Acinetobacter* spp., constitutes a great threat to public health and food safety (1). Carbapenem resistant *Acinetobacter baumannii* (CRAB) accounts for 53.7% among the *A. baumannii* isolates in 2020, China (2). About 78.2% of CRAB are isolated from ICUs, both adults and the elderly are more susceptible to *A. baumannii* (2). Environmental persistence and drug resistance enable the nosocomial thriving of *A. baumannii* (3). Due to the frequent acquisition of external genes related to antibiotic resistance and virulence, *A. baumannii* showed extensive stress tolerance to desiccation, antibiotics, and disinfectants (4). It is estimated that there are more than 45,000 infections in the United States, and one million cases globally per year caused by such pathogen (5). Recently, mucoid *A. baumannii* isolates, often associated with chronic infections, are multidrug resistant (MDR) with altered bacterial virulence (6). Besides, the increased blood derived isolates suggest the occurrence of phagocytic resistance in *A. baumannii* (7). Due to the elevated persistence, mucoid bacterial pathogens could not be eliminated by host immune systems, posing a threat to public health worldwide (8).

Due to the overproduction of capsular polysaccharide, mucoidity phenotype is an important adaptive defense response to the external pressure in pathogens (6, 9). Previous works have showed that matt (not glossy) *A. baumannii* strains evolve to the mucoid phenotype *in vivo*, and antibiotics such as chloramphenicol and erythromycin could promote the bacterial hypermucoid state (10, 11). Meanwhile, alterations between non-mucoid and mucoid phenotypes have also been reported in other pathogens such as *Pseudomonas aeruginosa* and *Klebsiella pneumoniae* under external stresses of antibiotics, oxygen deficiency, and immune response (12, 13). Moreover, mucoidity usually aggravates infections through regulating the increased expression of bacterial virulent factors. For example, hypermucoviscosity is a major phenotype associated with hypervirulence in *K. pneumoniae*, leading to invasive infections (metastatic dissemination) in adults (9, 14). Therefore, mucoidity promotes the survival of pathogens under harsh niches. Although certain mucoid related phenotypic characteristics have been elucidated, the relationship among mucoidity, virulence, and phagocytosis in *A. baumannii* remains unclear.

Macrophages play a pivotal role in exterminating bacterial pathogens, while many bacteria evolve adaptive strategies to circumvent the clearance of macrophages such as anti-phagocytosis (15, 16). For example, the negatively charged capsule is resistant to phagocytosis through the charge repulsion, resulting in the inhibition of alternative complement (17). These mucoid pathogens covered with capsule are anti-phagocytic, subsequently promoting the dissemination with increased mortality (8, 10). However, the underlying mechanism of mucoidity in anti-phagocytosis are poorly elucidated. A better understanding of the mucoidity in *A. baumannii* may shed light on the development of alternative interventions to minimize the potential impact of such pathogens.

In this study, we found mucoid *A. baumannii* strains were MDR and showed resistance to acid and peroxide exposure. Then we observed the hypermucoid strain resistance to the phagocytosis. The anti-phagocytic phenotype was associated with the high MW capsule through reducing the deposition of C3b.

## Results

### Mucoid phenotype, biofilm-forming ability, and toxicity of *Acinetobacter baumannii* isolates

**FIGURE 1:**
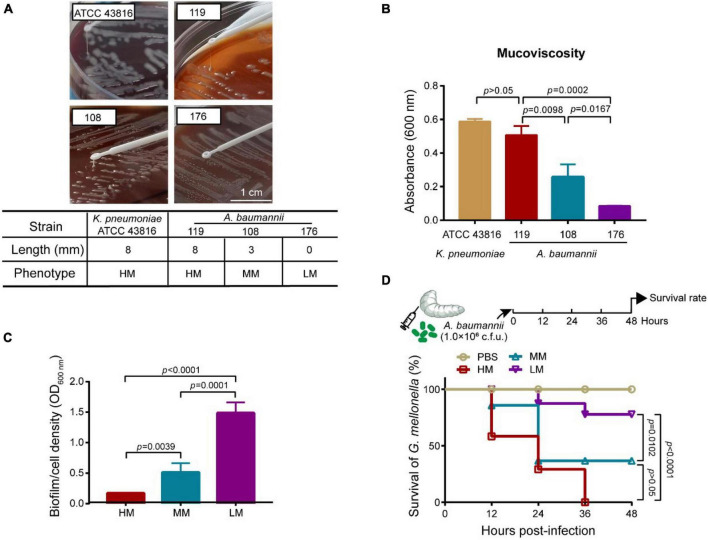
Mucoid phenotype, biofilm production and toxicity of mucoid *A. baumannii* strains. **(A)** Stretching of the colonies on an agar plate. Inserted table showed the length of viscous string and phenotype of isolates (bottom). *K. pneumoniae* ATCC 43816 was used for hypermucoviscous control. Scale bar = 1 cm. **(B)** Measurement of mucoviscosity of different strains. **(C)** Quantitative analysis of biofilm abundance in *A. baumannii* isolates at 24 h. Experiments in panels **(A–C)** were performed as three biologically independent experiments, and the mean ± SD was shown. *P* values were determined using an unpaired, two-tailed Student’s *t*-test. **(D)** Survival rates of *G. mellonella* larvae. Infected larvae (*n* = 7) with *A. baumannii* (1.0 × 10^6^CFU) at the right posterior gastropod. *P* values were determined using the two-sided, log[rank] (Mantel–Cox) test.

We analyzed the general biological characteristics of *A. baumannii* strains 119, 108, and 176 isolated from ICUs. These strains show perceptible differences in mucoid abundance by viscous string analysis (Figure 1A), classifying as hypermucoid (HM) (*A. baumannii* 119), medium mucoid (MM) (*A. baumannii* 108), and low mucoid (LM) (*A. baumannii* 176). The mucoviscosity were further confirmed based on the low-speed centrifugation method (Figure 1B). Besides, all three isolates were subject to the whole genome sequencing (WGS). Virulence factors of pathogenic bacteria (VFDB) analysis of the WGS data reveled the absence of *csuA/BABCDE* locus [relating to the capability of biofilm formation and immune evasion (18)] in the HM strain, which was consistent with the phenotype of poor biofilm-forming ability and high mortality to *Galleria mellonella* (Figures 1C,D and Supplementary Figure 1). However, although there is no resistance genes including *aac(6’)-lb-cr*, *msr(E)*, *mph(E)*, and *sul1/sul2*, the HM strain shows resistance to ciprofloxacin, clindamycin, erythromycin, and trimethoprim/sulfamethoxazole (Table 1, Supplementary Table 4, and Supplementary Figure 1A). It consists with the previous observation that decreased drug penetration contributes to antibiotic resistance in mucoid *A. baumannii* isolates (6). Taken together, the HM strain is MDR with poor biofilm-forming ability and high toxicity.

**TABLE 1:** Minimal inhibit concentration (MIC) values (μg/mL) of *A. baumannii* isolates.

Strains	β -Lactam			Aminoglycoside	Tetracycline	Fluoroquinolone	Polypeptide
	CAR	MER	CAZ	GEN	TET	CIP	COL
119	>128	128	128	4	>128	128	0.125
108	>128	128	128	>128	128	32	0.125
176	>128	>128	128	>128	>128	64	0.25
ATCC 17978	>128	128	128	>128	4	128	0.25
ATCC 19606	>128	64	128	>128	4	128	0.25
ATCC 25922	4	<0.03	0.25	1	2	<0.008	0.125

CAR, carbenicillin; MER, meropenem; CAZ, ceftazidime; GEN, gentamycin; TET, tetracycline; CIP, ciprofloxacin; COL, colistin. ATCC 17978, ATCC 19606, and ATCC 25922 were obtained from American Type Culture Collection, and the other bacteria tested are clinical isolates from a hospital in Zhejiang, China. *E. coli* ATCC 25922 was the standard quality control strains for AST tests. Additionally, A. *baumannii* ATCC 17978 and ATCC 19606 are reference strains.

### Mucoid *Acinetobacter baumannii* is resistant to acid and hydrogen peroxide

**FIGURE 2:**
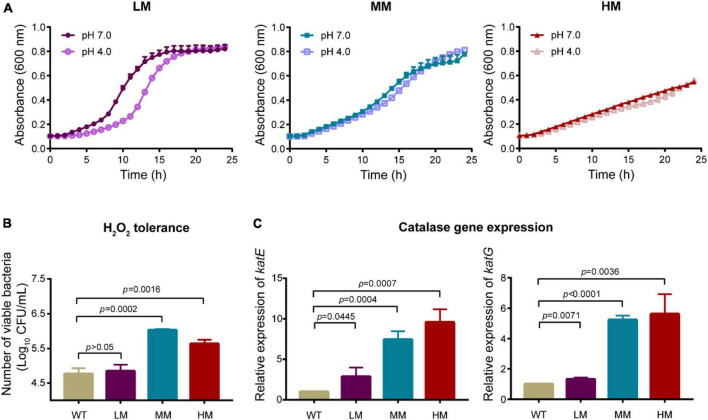
Mucoid *A. baumannii* shows tolerance to acid and hydrogen peroxide. **(A)** Growth dynamics of *A. baumannii* LM, MM, and HM strains under different pH conditions for 24 h. **(B)** H_2_O_2_ tolerance of *A. baumannii* LM, MM, and HM strains. *A. baumannii* strains were treated with 50 mmol/L H_2_O_2_ for 30 min. WT: *A. baumannii* ATCC 19606. **(C)** The mRNA expression of catalase genes *katE* and *katG* in WT and mucoid *A. baumannii* isolates. All experiments were performed as three biologically independent experiments, and the mean ± SD was shown. *P* values were determined using an unpaired, two-tailed Student’s *t*-test.

We evaluated the growth rate of LM, MM, and HM isolates under either acid or hydrogen peroxide (H_2_O_2_) conditions, respectively. Both the LM and the MM strains enter into the stationary phase after 20 h, whereas the LM strain shows a sharp logarithmic phase (Figure 2A). In contrast, the HM strain remains at the logarithmic growth phase at 24 h, which may be due to high metabolic cost of mucus production. Moreover, the LM strain is more sensitive to H_2_O_2_ than the MM strain (Figure 2B), consisting with the lower transcript levels of catalase associated genes *katE* and *katG* in LM than MM (Figure 2C). Meanwhile, the expression level of *katE* and *katG* are highly associated with the mucoid phenotype (Figure 2C). These results demonstrate that the mucoid *A. baumannii* isolates are tolerance to the exposure of either acid or hydrogen peroxide.

### Mucoid *Acinetobacter baumannii* shows anti-phagocytic phenotype

**FIGURE 3:**
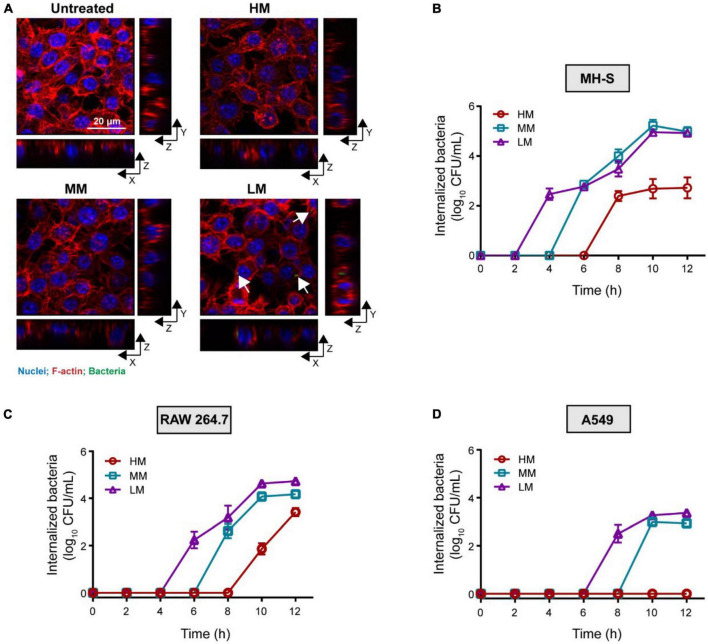
Hypermucoviscous *A. baumannii* shows anti-phagocytic phenotype. **(A)** Internalized *A. baumannii* in macrophages. MH-S cells are infected with *A. baumannii* (MOI = 10) for 4 h. Bacteria were labeled with pHrodo (green). F-actin and nuclei were labeled with rhodamine phalloidin (red) and DAPI (blue), respectively. Scale bar = 20 μm. **(B–D)** Viable counts of the internalized *A. baumannii* in MH-S cells **(B)**, RAW 264.7 **(C)**, and A549 cells **(D)**, infected with mucoid *A. baumannii* at MOI of 10 for 2–12 h. All experiments were performed as three biologically independent experiments, and the mean ± SD was shown.

To compare the invasion of these *A. baumannii* isolates, we co-cultured the strains with mouse lung macrophages (MH-S), mouse monocyte macrophages (RAW 264.7), and human alveolar basal epithelial cells (A549), respectively. Given that the growth of these strains shows no difference in cell culture media (Supplementary Figure 2), the LM, MM, and HM strains were first incubated with MH-S and RAW 264.7, respectively. Colistin (100 μg/mL) was used to eradicate the extracellular bacteria without causing cytotoxicity to mammalian cells (Supplementary Figure 3). The intracellular bacteria always appeared early in the macrophages infected with LM (Figures 3A–C), suggesting a positive relevance between mucoid phenotype and anti-phagocytic ability. The viable counts of internalized LM is higher than the others. Meanwhile, we excluded the explanation that the cytotoxicity of LM, MM, and HM to cells is response for such difference (Supplementary Figure 4). Moreover, we found that there is less intracellular HM in epithelial cells as well (Figure 3D). To further explore whether mucoid bacteria are resistance to phagocytosis, we examined the anti-phagocytic ability of *K. pneumoniae*. Compared to the low mucoid *K. pneumoniae* WNX-2, high mucoid *K. pneumoniae* ATCC 43816 hardly invade macrophages (Supplementary Figure 5), indicating a general behavior of anti-phagocytosis in pathogens with the mucoid phenotype. Altogether, we find that the mucoid *A.baumannii* shows anti-phagocytic activity to both macrophages and epithelial cells.

### Mucoid *Acinetobacter baumannii* reduces C3b deposition

**FIGURE 4:**
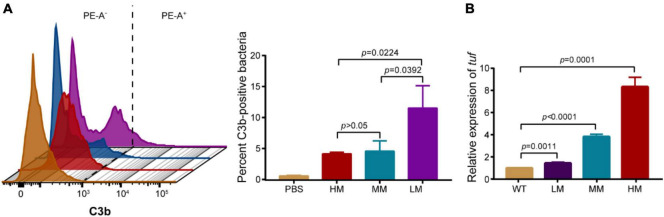
Hypermucoviscous *A. baumannii* reduces C3b deposition. **(A)** Representative histograms of anti-C3b fluorescence in *A. baumannii* **(left)**. Quantitative analysis of C3b-positive bacteria **(right)**. PE-A as the anti-C3b fluorophore. 10,000 events were collected per condition for flow cytometry, gated for singlets via FSC/SSC, fluorescence gate set to exclude 99% of isotype control and copied across samples ran in parallel. **(B)** The mRNA expression of translation elongation factor *tuf* in WT and mucoid *A. baumannii*. All experiments were performed as three biologically independent experiments, and the mean ± SD was shown. *P* values were determined using an unpaired, two-tailed Student’s *t*-test.

The increased C3b deposition on bacterial surface facilities phagocytosis through enhancing opsonization (19). We evaluated the relative abundance of C3b deposition on mucoid bacteria using flow cytometry. Results show that the strain with lower mucus has a higher abundance of C3b deposition among the LM, MM, and HM strains. C3b on LM was nearly three-time higher than the others (Figure 4A). Given that the HM and MM strains show no difference in C3b positive signals, we hypothesized that the consumption of C3b could also reduce its deposition on bacteria. Compared to the MM strain, the transcriptional level of translation elongation factor (*tuf*) (relating to C3b hydrolysis) is relatively high in the HM isolate (Figure 4B), indicating the greater consumption of C3b in HM (20). Besides, the similar content of lipooligosaccharide (LOS) in the LM, MM, and HM isolates, implied that the release of LOS is not a major cause of the different anti-phagocytic activities (21, 22). These results suggest that the HM strain shows potent anti-phagocytic activity through the reduction of C3b deposition and high potential of C3b hydrolysis.

### Capsule reduces the deposition of C3b

**FIGURE 5:**
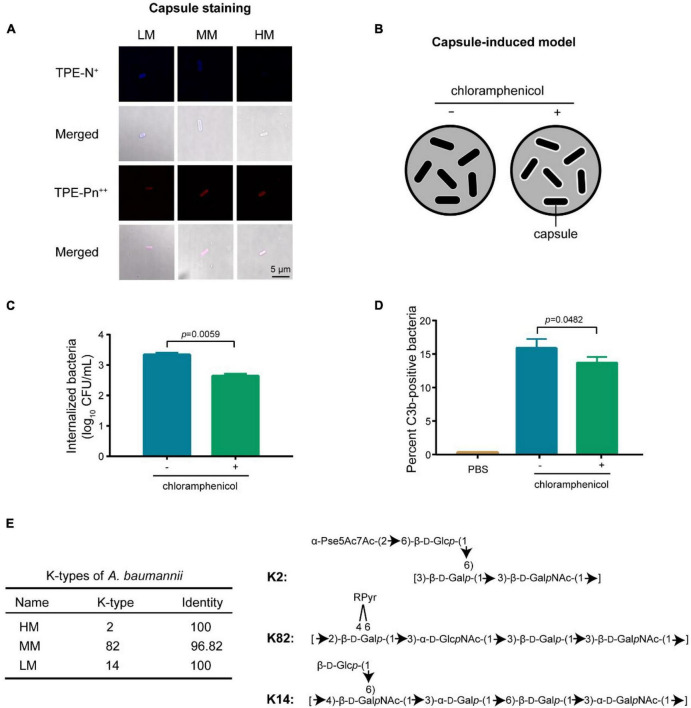
Capsular changes decreases the C3b deposition. **(A)** Confocal images of single charged probe TPE-N^+^ (20 μM) and zwitterionic probe TPE-Pn^++^ (20 μM). Scale bar = 5 μm. **(B)** Scheme of the introduction of hyper-production of capsule in *A. baumannii*. **(C)** CFUs of internalized bacteria in MH-S cells. Cells are infected with chloramphenicol-treated (10 μg/mL) *A. baumannii* ATCC 17978 at MOI of 10 for 4 h. **(D)** Quantitative analysis of C3b-positive bacteria by flow cytometry. Experiments in panels **(C,D)** were performed as three biologically independent experiments, and the mean ± s.d. was shown. *P* values were determined using an unpaired, two-tailed Student’s *t*-test. **(E)** Structural prediction of the repeating unit in capsule from mucoid *A. baumannii*.

Capsular polysaccharide mediates anti-phagocytic activities by reducing the C3b deposition on bacterial surface (23, 24). Therefore, we investigated the capsular difference in LM, MM, and HM using the zwitterionic TPE-Pn^++^ (with strong membrane-penetrating capability) and monocharged TPE-N^+^ (unable to stain bacteria with capsular) (25). Results indicate that the HM strain carries a thick capsule (Figure 5A and Supplementary Figure 7). The capsular differences are further evaluated using the alcian staining. The MM strain produces the highest amount of capsule, and the HM strain shows the highest molecular weight of the produced capsule (Supplementary Figure 11), indicating that the yield and composition of capsule varies in mucoid isolates.

The MM isolate has less C3b deposition with high capsular production, consisting with the decreased C3b deposition potential in high capsular strains (Figure 4). To further decipher the correlation between C3b deposition and capsular production, we introduced a capsular reversible and hyper-production *A. baumannii* model (Figure 5B). The inducing agent (chloramphenicol) has no cytotoxicity to macrophages (Supplementary Figures 8B, 9). We observed that the decreased C3b deposition potentiated the anti-phagocytic activity in capsular hyper-produced *A.baumannii* (Figures 5C,D).

The efficiency of C3b depositionis modulated by the capsular structure including the hydroxyl group and the backbone length of polysaccharide chain (26–28). According to the capsular classification database (27–29), the HM, MM, and LM isolates are classified as types of K2, K82, and K14 (Figure 5E), respectively. Compared to K82, K2 has shorter backbone while longer length of polysaccharide (10), resulting in a better anti-phagocytic activity through effectively inhibiting the deposition of C3b (Figure 3). Altogether, the abundance and composition of capsule reduces the deposition of C3b, resulting an anti-phagocytic activity in the hyper mucoid *A. baumannii*.

## Discussion

In the present study, we explored the biological characteristics of clinical ICUs derived *A. baumannii* strains. Results show that the hyper mucoid isolate is MDR with high tolerance to the exposure of either acid or hydrogen peroxide. Besides, the HM strain exhibits greater anti-phagocytic to both macrophages and epithelial cells than the LM strain. Further analysis reveals that the enhanced anti-phagocytosis is related to the reduction of C3b deposition in mucoid *A. baumannii*.

Due to the lack of *csuA/BABCDE* locus (relating to biofilm formation) (30), the HM strain exhibits poor biofilm-forming ability *in vitro*. However, the phenotype of MDR and virulence are inconsistent with the genotypes in mucoid *A. baumannii*. Though no relevant drug-resistance genes are sequenced, the HM strain shows MDR due to the poor penetration of antibiotics on the mucoid bacterial surface (10). Such phenotype has been reported in *H. pylori*, *P. aeruginosa*, and *A. baumannii* (31–35). Furthermore, compared to the same virulence-associated genes in LM strain, the HM strain shows high mortality in the *G. mellonella* infection model. Last, the hyper mucoid *A. baumannii* shows the elevated molecular weight in capsular polysaccharide, reduced C3b deposition, and enhanced anti-phagocytic activities. Previous works demonstrate that the capsular polysaccharide prevents the C3b mediated phagocytosis in mammal cells (26, 28, 36). It suggests a potential relationship between capsule and mucus in *A. baumannii* (9, 37–39), however, the underlying mechanism remains unclear.

Mucoid phenotype is a crucial defense response under external stresses for bacterial pathogens (6, 8, 10, 40). Generally, hyper mucoid isolates with enhanced anti-phagocytic activity often cause persistent blood infections (8, 10, 35). Capsular polysaccharide targeted therapeutic approaches might facilitate bacterial clearance by elevating the opsonic activity of host cells, providing a novel insight to the treatment of mucoid pathogens associated infections.

## Materials and methods

### Bacterial strains and mammalian cells

All bacterial strains used in this study were listed in Supplementary Table 1. Routinely, bacteria were cultured at 37°C in brain heart infusion (BHI) (Beijing Land Bridge Technology, Shanghai, China) medium with shaking at 200 rpm (revolution per minute). A549 and RAW 264.7 cells (Supplementary Table 2) were cultured in Dulbecco’s modified Eagle’s medium (DMEM) (Thermo Fisher Scientific, Waltham, MA, United States). MH-S cells (Supplementary Table 2) were cultured in Roswell Park Memorial Institute (RPMI)-1640 medium (Thermo Fisher Scientific, Waltham, MA, United States). All the media were supplemented with 10% heat inactivated fetal bovine serum (FBS) (Invitrogen, Thermo Fisher Scientific, Waltham, MA, United States) and 1% (w/v) penicillin-streptomycin (Solarbio Life Science, Shanghai, China).

### String test and mucoviscosity assay

The *A. baumannii* isolates were evaluated by string test as described previously (41). All tested strains were cultured on sheep blood agar plate (5%) overnight at 37°C, then a single bacterial colony was stretched with an inoculation loop. The mucoviscosity assay was performed by low-speed centrifugation (39). Briefly, the tested strains were incubated in Luria-Bertani broth (LB) (Beijing Land Bridge Technology, Shanghai, China) at 37°C with shaking overnight. Then cultures were centrifuged at 1,000 *g* for 5 min. The absorption of supernatant was measured under the wavelength of 600 nm using an Infinite M200 Microplate reader (Tecan).

### Biofilm-forming assay

The biofilm abundance was detected following a previously described method with some modifications (42). Briefly, 100 μL of 1 × 10^6^ CFUs/mL *A. baumannii* strains was cultured in Mueller–Hinton broth (MHB) (Land Bridge Technology, Beijing, China) at 37°C for 24 h. The densities of bacteria transferred to new wells were measured under the wavelength of 600 nm by Infinite M200 Microplate reader. Then the original wells were washed three times with sterile phosphate buffered saline (PBS), following the air-drying, and 1% crystal violet stanning. The bound dye was resolubilized in 95% ethanol and the absorption of the dye solution was measured under the wavelength of 600 nm by Infinite M200 Microplate reader.

### *In vivo* toxicity test

The virulence of *A. baumannii* isolates were evaluated *in vivo* using the *G. mellonella* larvae infection model as previously described (43). The healthy larvae (0.25–0.35 g) of *G. mellonella* (purchased from Huiyude Biotech Company, Tianjin, China) were randomly divided into four groups (*n* = 7 per group) and infected with 10 μL of *A. baumannii* strains suspension (1.0 × 10^6^CFUs) at the right pleopod and the other groups were injected with an equal volume of PBS. Survival rates of *G. mellonella* were recorded for 2 days.

### Antibacterial susceptibility test, acid resistance, and H_2_O_2_ tolerance

The antibacterial susceptibility test was performed by broth microdilution according to the Clinical and Laboratory Standards Institute (CLSI) guideline (44). Briefly, antibiotics were two-fold diluted in MHB and mixed with an equal volume of bacterial suspensions in MHB containing approximately 1.5 × 10^6^ CFUs/mL in a clear, UV-sterilized, 96-well plate. After 16–20 h incubation at 37°C, minimal inhibit concentration (MIC) were defined as the lowest concentrations of antibiotics with no visible growth of bacteria.

For acid tolerance assay, fresh prepared bacteria were dilution by 1:100 in LB broth (pH 7.0), mixed with an equal volume of pH 4.0 medium in a 96-well microplate. The growth dynamics were recorded under the wavelength of 600 nm with an interval of 1h at 37°C measured by Infinite M200 Microplate reader.

Fresh prepared cultures were adjusted to McFarland turbidity of 0.5 and diluted in 4 mL BHI broth. The bacterial suspensions were treatment with 50 mmol/L H_2_O_2_ (Sinopharm Chemical Reagent Co., Shanghai, China) for 30 min, following by plating serial dilution on BHI agar plates. Then the CFUs were counted after incubating at 37°C for 24 h.

### Confocal laser scanning microscopy analysis

MH-S cells were plated on glass coverslips (14 mm, NEST Life and Science Technology Co., Wuxi, China) in 24-well culture plates to form monolayers. Then the cells were infected with pHrodo Green-labeled (Invitrogen, Thermo Fisher Scientific, Waltham, MA, United States) *A. baumannii* strains [multiplicity of infection (MOI) = 10] for 4 h, following fixating in 4% paraformaldehyde for 20 min. F-actin and nuclei were labeled with ActinRed^555^ ReadyProbes (Invitrogen, Thermo Fisher Scientific, Waltham, MA, United States) and DAPI (Beyotime Biotechnology, Shanghai, China), respectively. Images were captured using a Leica SP8 confocal microscope, and Z-axis sections were cut every 3 μm to analyze the location of internalized bacteria. Images were analyzed and merged by LAS AF Lite software (Leica Biosystems, Germany).

Bacterial imaging assay was performed as described previously (25). Specifically, fresh prepared bacterial cultures were washed and resuspended with 200 μL PBS. Then the bacterial solutions were transferred into a sterilized EP tube with probe solutions (20 μmol/L) and incubated at room temperature for 30 min. After that, 10 μL of the stained bacteria was transferred to a piece of clean glass slide and then covered by a coverslip for fixation. Images were collected and analyzed by LAS AF Lite software.

### Cell infection

The cell infection assay was performed as described previously, with some modifications (45). Mammalian cells with 4 × 10^5^ were seeded at 24-well plates to form monolayers. Then, bacterial resuspensions were diluted in DMEM or RPMI-1640 medium supplemented with 1% FBS and cocultured with cells at an MOI of 10. At the end of the trials, cells were incubated for an additional 30 min with 100 μg/mL colistin to remove the extracellular bacteria. After washing with PBS, the cells were lysed by DMEM or RPMI-1640 medium supplemented with 0.1% Triton-X 100 (Beyotime Biotechnology Co., Shanghai, China). The harvested bacteria were plating on BHI agar plates with different dilutions for the Colony-count technique to quantify the number of internalized bacteria. In the capsule-induced model, chloramphenicol (10 μg/mL) was added to the bacterial suspensions for the stress maintenance.

### Capsule extracting and staining

Extraction of *A. baumannii* capsule was performed as described previously with some modifications (46). Briefly, cultures were resuspended with 200 μL lysis buffer (60 mmol/L Tris, pH8; 10 mmol/L MgCl_2_; 50 μml/L CaCl_2_; 20 μg/mL DNase and RNase; and 3 mg/mL lysozyme), then incubated at 37°C for 1 h. Following vortex and three repeated liquid nitrogen/37°C freeze-thaw cycles, additional DNase and RNase were added and incubated at 37°C for 30 min. About 10 μL 10% SDS was then added and incubated at 37°C for another 30 min. The suspensions were boiled at 100°C for 10 min and then incubated at 60°C with protease K for 1 h. After centrifugation, the supernatants were retained and precipitated overnight in pre-cooling 75% ethanol, followed by pelleting, air-drying, resuspending with SDS sample buffer at a volume normalized based on OD_600_ and boiling for 5 min.

Samples were separated on 4–20% BioRad TGX Tris-glycine gels (Bio-RAD, Hercules, CA, United States). After electrophoresis, the gel was washed with deionized water and stained with a solution of 0.1% (w/v) of Alcian Blue 8GX (Sigma-Aldrich, Merck, Germany) for 1 h. Gels were decolorized by placing in a pH 4.75 solution containing 40% ethanal and 60% 20 mmol/L sodium acetate for overnight.

### Capsule-induced model

Method of capsule induction was performed as described previously, with some modifications (10). About 10 μg/mL chloramphenicol was added to logarithmic phase bacteria. After overnight incubation, the capsule of *A. baumannii* strains was extracted and analyzed with alcian blue staining.

### C3b deposition assay

For quantifying the C3b deposition, previously described method was used with some modification (47). Briefly, *A. baumannii* isolates were cultured overnight and adjusted to McFarland turbidity 0.5, then 100 μL bacterial suspension was mixed with 100 μL mouse serum and incubated at 37°C for 30 min. After PBS washing, samples were incubated with antibodies against mouse complement factor C3b (Thermo Fisher Scientific, United States) and incubated with a secondary fluorescent antibody for another 30 min subsequently. Samples were then resuspended with PBS and analyzed using Becton-Dickinson FACS Canto II flow cytometer. The gating on single cells with positive gates established at a fluorescence excluding 99% of the isotype control samples.

### RT-qPCR analysis

Bacterial total RNA was extracted and examined using M5 EASYspin Plus kit (Mei5bio, Beijing, China) and Nanodrop spectrophotometer (Thermo Scientific, MA, United States), respectively. Reverse transcription was performed using a PrimeScript RT reagent Kit with gDNA Eraser (Takara, Beijing, China) with the manufacturer’s protocol. The messenger RNA levels relative to those of the control genes 16S were determined by real-time PCR tests with PowerUp SYBR Green Master Mix (Applied Biosystems, Thermo Fisher Scientific, Carlsbad, CA, United States). RT-PCR tests were performed using the ABI Quantstudio 7 detection system (Applied Biosystems, Thermo Fisher Scientific, Carlsbad, CA, United States). The fold changes in gene expression were determined using the 2^–ΔΔCt^ method. Primers used in this study were listed in Supplementary Table 3.

### K-typing analysis

As previously described (48), the capsular K-type of related *A. baumannii* isolates were analyzed using *wzc* gene BLAST.

### Statistical analysis

Statistical analysis was performed using GraphPad Prism 7.0 (GraphPad Software, Inc.). All data were expressed as the mean ± SD and unless otherwise noted, unpaired t-test between two groups were used to calculate *p*-values.

## Data availability statement

The original contributions presented in this study are included in the article/Supplementary material, further inquiries can be directed to the corresponding author.

## Ethics statement

The studies involving human participants were reviewed and approved by Research Ethics Committee of the Second Affiliated Hospital of Zhejiang University. The patients/participants provided their written informed consent to participate in this study. The animal study was reviewed and approved by Research Ethics Committee of the Second Affiliated Hospital of Zhejiang University.

## Author contributions

XG: methodology, validation, and data curation. QZ: formal analysis and validation. YW: data analysis and validation. HZ: resources and data curation. SD: data analysis and supervision. KZ: conceptualization, project administration, and data analysis. XG, QZ, and KZ: writing the manuscript. All authors contributed to the article and approved the submitted version.

## References

[1] He T Wang R Liu D Walsh TR Zhang R Lv Y Emergence of plasmid-mediated high-level tigecycline resistance genes in animals and humans. *Nat Microbiol.* (2019) 4:1450–6. 10.1038/s41564-019-0445-2 31133751

[2] China Antimicrobial Resistance Surveillance System [CARSS]. *National Bacterial Resistance Surveillance Report (Abbreviated Version).* (2020). Available online at: http://www.carss.cn/Report/Details/808 (accessed March 18, 2022).

[3] Harding CM Hennon SW Feldman MF. Uncovering the mechanisms of *Acinetobacter baumannii* virulence. *Nat Rev Microbiol.* (2018) 16:91–102. 10.1038/nrmicro.2017.148 29249812 PMC6571207

[4] Wong D Nielsen TB Bonomo RA Pantapalangkoor P Luna B Spellberg B. Clinical and pathophysiological overview of *Acinetobacter* infections: a century of challenges. *Clin Microbiol Rev.* (2017) 30:409–47. 10.1128/cmr.00058-16 27974412 PMC5217799

[5] Spellberg B Rex JH. The value of single-pathogen antibacterial agents. *Nat Rev Drug Discov.* (2013) 12:963. 10.1038/nrd3957-c1 24232373 PMC4012226

[6] Shan W Zhang H Kan J Yin M Zhang J Wan L Acquired mucoid phenotype of *Acinetobacter baumannii*: impact for the molecular characteristics and virulence. *Microbiol Res.* (2021) 246:126702. 10.1016/j.micres.2021.126702 33465557

[7] Aboshakwa AM Lalla U Irusen EM Koegelenberg CFN. *Acinetobacter baumannii* infection in a medical intensive care unit: the impact of strict infection control. *Afr J Thorac Crit Care Med.* (2019) 25:10.7196/AJTCCM.2019.v25i1.239. 10.7196/AJTCCM.2019.v25i1.239 34286240 PMC8278987

[8] Ernst CM Braxton JR Rodriguez-Osorio CA Zagieboylo AP Li L Pironti A Adaptive evolution of virulence and persistence in carbapenem-resistant *Klebsiella pneumoniae*. *Nat Med.* (2020) 26:705–11. 10.1038/s41591-020-0825-4 32284589 PMC9194776

[9] Mike LA Stark AJ Forsyth VS Vornhagen J Smith SN Bachman MA A systematic analysis of hypermucoviscosity and capsule reveals distinct and overlapping genes that impact *Klebsiella pneumoniae* fitness. *PLoS Pathog.* (2021) 17:e1009376. 10.1371/journal.ppat.1009376 33720976 PMC7993769

[10] Geisinger E Isberg RR. Antibiotic modulation of capsular exopolysaccharide and virulence in *Acinetobacter baumannii*. *PLoS Pathog.* (2015) 11:e1004691. 10.1371/journal.ppat.1004691 25679516 PMC4334535

[11] Hua X Zhou Z Yang Q Shi Q Xu Q Wang J Evolution of *Acinetobacter baumannii* in vivo: international clone II, more resistance to ceftazidime, mutation in ptk. *Front Microbiol.* (2017) 8:1256. 10.3389/fmicb.2017.01256 28740486 PMC5502287

[12] Chiarelli A Cabanel N Rosinski-Chupin I Zongo PD Naas T Bonnin RA Diversity of mucoid to non-mucoid switch among carbapenemase-producing *Klebsiella pneumoniae*. *BMC Microbiol.* (2020) 20:325. 10.1186/s12866-020-02007-y 33109078 PMC7590720

[13] Winstanley C O’Brien S Brockhurst MA. *Pseudomonas aeruginosa* evolutionary adaptation and diversification in cystic fibrosis chronic lung infections. *Trends Microbiol.* (2016) 24:327–37. 10.1016/j.tim.2016.01.008 26946977 PMC4854172

[14] Catalán-Nájera JC Garza-Ramos U Barrios-Camacho H. Hypervirulence and hypermucoviscosity: two different but complementary *Klebsiella* spp. phenotypes. *Virulence.* (2017) 8:1111–23. 10.1080/21505594.2017.1317412 28402698 PMC5711391

[15] Liu X Wu Y Mao C Shen J Zhu K. Host-acting antibacterial compounds combat cytosolic bacteria. *Trends Microbiol.* (2022) 30:761–77. 10.1016/j.tim.2022.01.006 35140036

[16] Nagre N Cong X Terrazas C Pepper I Schreiber JM Fu H Inhibition of macrophage complement receptor CRIg by TRIM72 polarizes innate immunity of the lung. *Am J Respir Cell Mol Biol.* (2018) 58:756–66. 10.1165/rcmb.2017-0236OC 29268030 PMC6002657

[17] Cress BF Englaender JA He W Kasper D Linhardt RJ Koffas MA. Masquerading microbial pathogens: capsular polysaccharides mimic host-tissue molecules. *FEMS Microbiol Rev.* (2014) 38:660–97. 10.1111/1574-6976.12056 24372337 PMC4120193

[18] Alvestegui A Olivares-Morales M Munoz E Smith R Nataro JP Ruiz-Perez F TLR4 participates in the inflammatory response induced by the AAF/II fimbriae from enteroaggregative *Escherichia coli* on intestinal epithelial cells. *Front Cell Infect Microbiol.* (2019) 9:143. 10.3389/fcimb.2019.00143 31131263 PMC6509964

[19] Heesterbeek DAC Angelier ML Harrison RA Rooijakkers SHM. Complement and bacterial infections: from molecular mechanisms to therapeutic applications. *J Innate Immun.* (2018) 10:455–64. 10.1159/000491439 30149378 PMC6784045

[20] Koenigs A Zipfel PF Kraiczy P. Translation elongation factor Tuf of *Acinetobacter baumannii* is a plasminogen-binding protein. *PLoS One.* (2015) 10:e0134418. 10.1371/journal.pone.0134418 26230848 PMC4521846

[21] Hao DJ Liu C Zhang L Chen B Zhang Q Zhang R Lipopolysaccharide and curcumin co-Stimulation potentiates olfactory ensheathing cell phagocytosis via enhancing their activation. *Neurotherapeutics.* (2017) 14:502–18. 10.1007/s13311-016-0485-8 27743319 PMC5398976

[22] Uronen-Hansson H Steeghs L Allen J Dixon G Osman M Ley P Human dendritic cell activation by *Neisseria meningitidis*: phagocytosis depends on expression of lipooligosaccharide (LOS) by the bacteria and is required for optimal cytokine production. *Cell Microbiol.* (2004) 6:625–37. 10.1111/j.1462-5822.2004.00387 15186399

[23] Kuipers A Stapels DAC Weerwind LT Ko YP Ruyken M Lee JC The *Staphylococcus aureus* polysaccharide capsule and Efb-dependent fibrinogen shield act in concert to protect against phagocytosis. *Microbiology (Read).* (2016) 162:1185–94. 10.1099/mic.0.000293 27112346 PMC4977062

[24] Sharma S Bhatnagar R Gaur D. *Bacillus anthracis* poly-γ-D-glutamate capsule inhibits opsonic phagocytosis by impeding complement activation. *Front Immunol.* (2020) 11:462. 10.3389/fimmu.2020.00462 32296419 PMC7138205

[25] Ai W Yang Z Ma Y Han X Chen Y Zhu K Combined tetraphenylethylene fluorogens with positive charge for imaging capsule-covered pathogens. *Analyst.* (2020) 145:6435–40. 10.1039/d0an00349b 32760975

[26] Berends ET Kuipers A Ravesloot MM Urbanus RT Rooijakkers SH. Bacteria under stress by complement and coagulation. *FEMS Microbiol Rev.* (2014) 38:1146–71. 10.1111/1574-6976.12080 25065463

[27] Sahu A Kozel TR Pangburn MK. Specificity of the thioester-containing reactive site of human C3 and its significance to complement activation. *Biochem J.* (1994) 302:429–36. 10.1042/bj3020429 8092994 PMC1137246

[28] Wilson RP Winter SE Spees AM Winter MG Nishimori JH Sanchez JF The Vi capsular polysaccharide prevents complement receptor 3-mediated clearance of *Salmonella enterica* serotype typhi. *Infect Immun.* (2011) 79:830–7. 10.1128/IAI.00961-10 21098104 PMC3028862

[29] Singh JK Adams FG Brown MH. Diversity and function of capsular polysaccharide in *Acinetobacter baumannii*. *Front Microbiol.* (2018) 9:3301. 10.3389/fmicb.2018.03301 30687280 PMC6333632

[30] Moon KH Weber BS Feldman MF. Subinhibitory concentrations of trimethoprim and sulfamethoxazole prevent biofilm formation by *Acinetobacter baumannii* through inhibition of csu pilus expression. *Antimicrob Agents Chemother.* (2017) 61(9):e778–17. 10.1128/aac.00778-17 28674047 PMC5571315

[31] Jones CJ Wozniak DJ. *Pseudomonas aeruginosa* contributes to the establishment of biofilms and immune evasion. *mBio.* (2017) 8:e864-17. 10.1128/mBio.00864-17 28634241 PMC5478896

[32] Hu L Shi Y Xu Q Zhang L He J Jiang Y Capsule thickness, not biofilm formation, gives rise to mucoid *Acinetobacter baumannii* phenotypes that are more prevalent in long-term infections: a study of clinical isolates from a hospital in China. *Infect Drug Resist.* (2020) 13:99–109. 10.2147/idr.S230178 32021324 PMC6957007

[33] Kadkhodaei S Siavoshi F Noghabi KA. Mucoid and coccoid *Helicobacter pylori* with fast growth and antibiotic resistance. *Helicobacter.* (2020) 25:e12678. 10.1111/hel.12678 31880001

[34] Malhotra S Limoli DH English AE Parsek MR Wozniak DJ. Mixed communities of mucoid and nonmucoid *Pseudomonas aeruginosa* exhibit enhanced resistance to host antimicrobials. *mBio.* (2018) 9:e275-18. 10.1128/mBio.00275-18 29588399 PMC5874919

[35] Tuchscherr L Medina E Hussain M Volker W Heitmann V Niemann S *Staphylococcus aureus* phenotype switching: an effective bacterial strategy to escape host immune response and establish a chronic infection. *EMBO Mol Med.* (2011) 3:129–41. 10.1002/emmm.201000115 21268281 PMC3395110

[36] Hostetter MK. Serotypic variations among virulent *Pneumococci* in deposition and degradation of covalently bound C3b - implications for phagocytosis and antibody-production. *J Infect Dis.* (1986) 153:682–93. 10.1093/infdis/153.4.682 3950449

[37] Petruzzi B Briggs RE Tatum FM Swords WE De Castro C Molinaro A Capsular polysaccharide interferes with biofilm formation by *Pasteurella multocida* serogroup A. *mBio.* (2017) 8:e1843-17. 10.1128/mBio.01843-17 29162713 PMC5698555

[38] Walker KA Miner TA Palacios M Trzilova D Frederick DR Broberg CA A *Klebsiella pneumoniae* regulatory mutant has reduced capsule expression but retains hypermucoviscosity. *mBio.* (2019) 10:e89-19. 10.1128/mBio.00089-19 30914502 PMC6437046

[39] Walker KA Treat LP Sepúlveda VE Miller VL. The small protein RmpD drives hypermucoviscosity in *Klebsiella pneumoniae*. *mBio.* (2020) 11:e1750-20. 10.1128/mBio.01750-20 32963003 PMC7512549

[40] Germoni LA Bremer PJ Lamont IL. The effect of alginate lyase on the gentamicin resistance of *Pseudomonas aeruginosa* in mucoid biofilms. *J Appl Microbiol.* (2016) 121:126–35. 10.1111/jam.13153 27061817

[41] Park S Lee H Shin D Ko KS. Change of *Hypermucoviscosity* in the development of tigecycline resistance in hypervirulent *Klebsiella pneumoniae* sequence type 23 strains. *Microorganisms.* (2020) 8:1562. 10.3390/microorganisms8101562 33050506 PMC7601201

[42] Mwangi J Yin Y Wang G Yang M Li Y Zhang Z The antimicrobial peptide ZY4 combats multidrug-resistant *Pseudomonas aeruginosa* and *Acinetobacter baumannii* infection. *Proc Natl Acad Sci USA.* (2019) 116:26516–22. 10.1073/pnas.1909585117 31843919 PMC6936460

[43] Li G Shi JM Zhao Y Xie YZ Tang Y Jiang XF Identification of hypervirulent *Klebsiella pneumoniae* isolates using the string test in combination with galleria mellonella infectivity. *Eur J Clin Microbiol Infect Dis.* (2020) 39:1673–9. 10.1007/s10096-020-03890-z 32318968

[44] Clinical Laboratory Standards Institute [CLSI]. *Performance Standards For Antimicrobial Susceptibility Testing.* Wayne, PA: Clinical Laboratory Standards Instidute (2021).

[45] Qu S Huang X Song X Wu Y Ma X Shen J A rigid nanoplatform for precise and responsive treatment of intracellular multidrug-resistant bacteria. *Engineering*. (2022) 15:8. 10.1016/j.eng.2021.12.021

[46] Tipton KA Rather PN. Extraction and visualization of capsular polysaccharide from *Acinetobacter baumannii*. In: Biswas I Rather PN editors. *Acinetobacter baumannii* Methods and Protocols: Methods and Protocols. New Jersey: Humana Press (2019). p. 227–31. 10.1007/978-1-4939-9118-1_21 30798559

[47] Talyansky Y Nielsen TB Yan J Carlino-Macdonald U Di Venanzio G Chakravorty S Capsule carbohydrate structure determines virulence in *Acinetobacter baumannii*. *PLoS Pathog.* (2021) 17:e1009291. 10.1371/journal.ppat.1009291 33529209 PMC7880449

[48] Hsieh YC Wang SH Chen YY Lin TL Shie SS Huang CT Association of capsular types with carbapenem resistance, disease severity, and mortality in *Acinetobacter baumannii*. *Emerg Microbes Infect.* (2020) 9:2094–104. 10.1080/22221751.2020.1822757 32912064 PMC7534287

