# Supplementary material for: Mucoid Acinetobacter baumannii enhances anti-phagocytosis through reducing C3b deposition
Source: Front Med (Lausanne). 2022 Sep 15;9:879361. doi: 10.3389/fmed.2022.879361 (PMC9521736; doi:10.3389/fmed.2022.879361)
Supplement: Supplementary file 1 [file Presentation_1.pdf]

Supporting information

Table S1 Bacterial strains used in this study.

**Strain**	**Description**
A. baumannii 119	A clinical isolate from human, hyper mucoid
A. baumannii 108	A clinical isolate from human, medium mucoid
A. baumannii 176	A clinical isolate from human, low mucoid
A. baumannii 12-1	Strain isolates from effective micro-organisms powder
A. baumannii 34	Strain isolates from porcine
A. baumannii ATCC 19606	Reference strain of *A. baumannii*
A. baumannii ATCC 17978	Reference strain of *A. baumannii*
K. pneumoniae ATCC 43816	Reference strain of *K. pneumoniae*, hyper mucoid
K. pneumoniae WNX-2	A clinical isolate from human, low mucoid
E.coli ATCC 25922	Reference strain of Gram-negative bacteria for MIC test

Table S2 Mammalian cells used in this work.

Cell line	Description
A549	Human lung carcinoma epithelial cells
MH-S	Mouse lung macrophages
RAW 264.7	Mouse monocyte macrophages

Table S3 RT-qPCR primers used in this study.

Primer	Sequences
wzc	F: CTCCACCAGTGCTTGCAGTA<br>R: CAGCGCTAGCACGTTGAATA
galU	F: AGCCAAGCTGCTCAAATCAT<br>R: CGGCCAACCACAGATAAGTT
katE	F: AACTTTGACTTCGATTTGCTGGA<br>R: TGTATGAAAATAGACGGGCTTGT
katG	F: GGCGATGAAAAAGAATGGTTA<br>R: ATTTCTTCATCATCCATTGCC
tuf	F: CACGCCGACTACGTTAAAAA<br>R: TACACCTACCTGACGAGAAA
16S	F: CAGCTCGTGTCGTGAGATGT<br>R: CGTAAGGGCCATGATGACTT

Table S4 Antimicrobial susceptibility profiles of *A. baumannii* isolates

	Macrolide		Sulphonamide	Lincosamide
	ERY	TIL	TRI/SUL	CLI
A. baumannii 119	64	>128	4/76	>128
A. baumannii 108	>128	>128	2/38	64
A. baumannii 176	>128	>128	2/38	64
E.coli ATCC 25922	32	128	0.5/9.5	32

MICs of antibiotics in μg/mL; ERY, erythromycin; TIL, tilmicosin; TRI/SUL, trimethoprim/sulfamethoxazole; CLI, clindamycin.

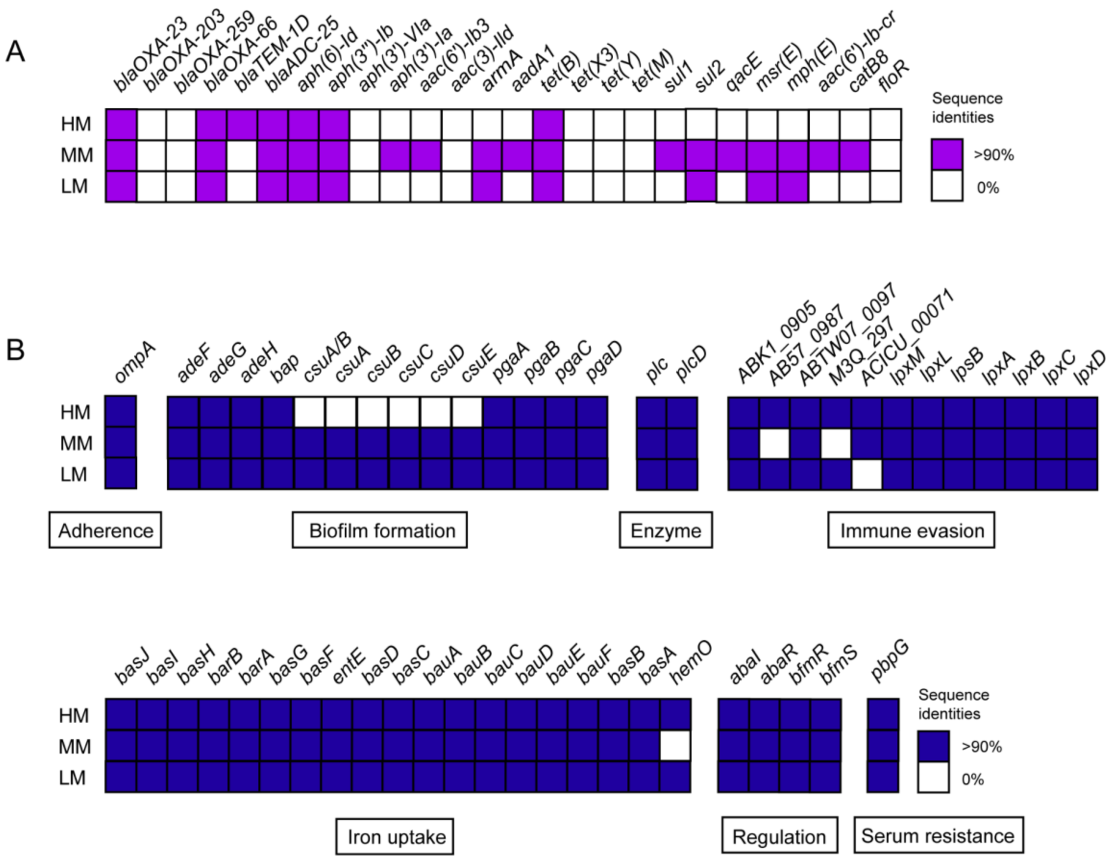

Figure S1 Distributions of antibiotic resistance and virulence associated genes in the *A. baumannii* strains. (A) Antibiotic resistance genes in the LM, MM, and HM strains; (B) Virulence genes in the LM, MM, and HM strains

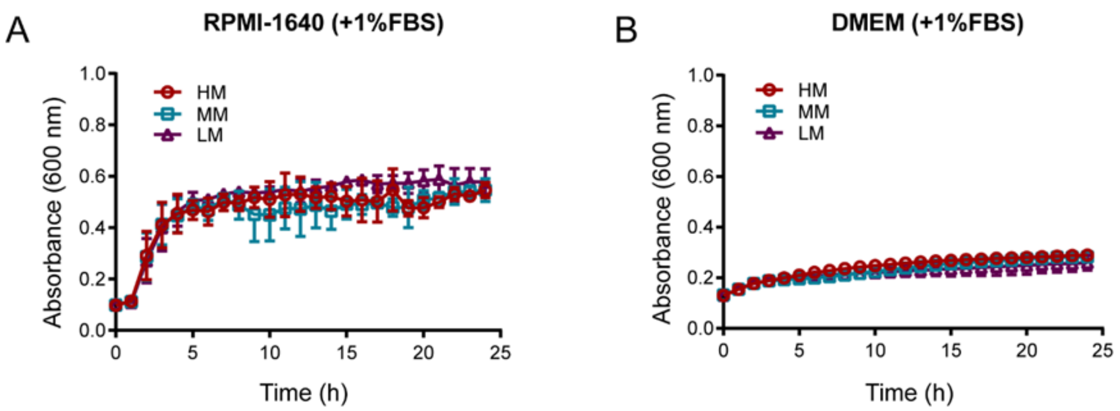

Figure S2 Similar growth dynamics of the LM, MM, and HM strains in RPMI-1640 and DMEM. (A) Growth dynamics of mucoid *A. baumannii* strains in RPMI-1640 media for 24 h. (B) Growth dynamics of mucoid *A. baumannii* strains in DMEM media for 24 h. All experiments were performed as three biologically independent experiments, and the mean ± s.d. was shown. *P* values were determined using an unpaired, two-tailed Student's *t*-test.

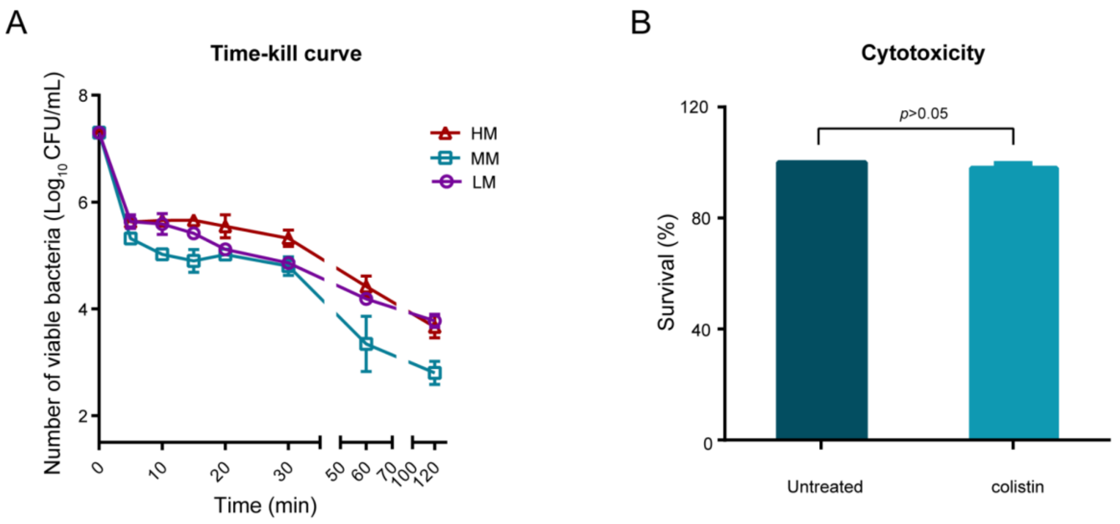

Figure S3 Time- killing curves to the *A.baumannii* LM, MM, and HM strains and cellular cytotoxicity of colistin. (A) Time-killing curves of colistin against *A. baumannii* strains *in vitro*. (B) Cytotoxicity of colistin (100 μg/mL) to MH-S cells. All experiments were performed as three biologically independent experiments, and the mean ± s.d. was shown. *P* values were determined using an unpaired, two-tailed Student's *t*-test.

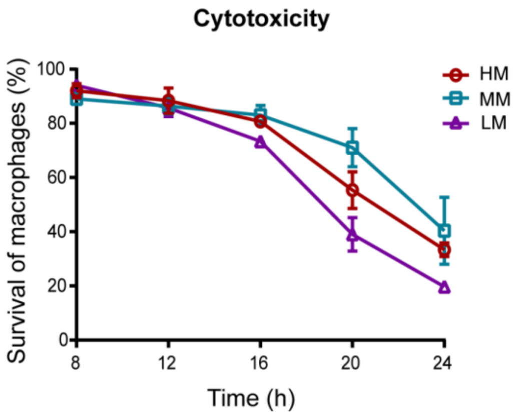

Figure S4 Cytotoxicity of the *A. baumannii* isolates to MH-S cells. Cytotoxicity examinations were performed on MH-S cells using a lactate dehydrogenase (LDH) cytotoxicity assay detection kit following the manufacturer's instructions. MH-S cells are infected with *A.baumannii* (MOI=10) for 8 to 24 h. The absorption of released lactate dehydrogenase (LDH) in the supernatant was evaluated. The experiment was performed as three biologically independent experiments, and the mean ± s.d. was shown.

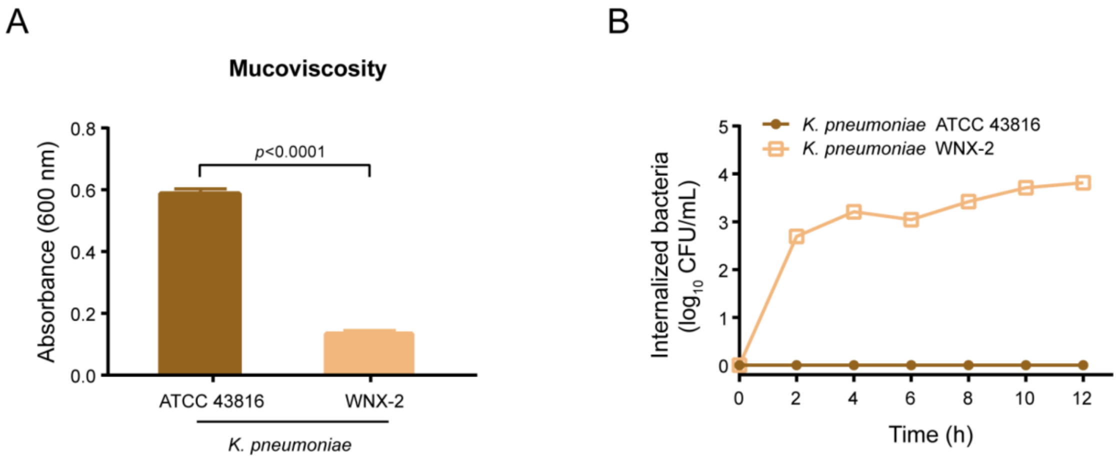

Figure S5 Mucoid *K. pneumoniae* ATCC 43816 shows anti-phagocytic activity. (A) Determination of mucoviscosity of *K. pneumoniae* strains. The experiment was performed as three biologically independent experiments, and the mean ± s.d. was shown. *P* values were determined using an unpaired, two-tailed Student's *t*-test. (B) CFUs of internalized *K. pneumoniae* strains in MH-S cells. MH-S cells are infected with *A.baumannii* (MOI=10) for 2 to 12 h. All experiments were performed as three biologically independent experiments, and the mean ± s.d. was shown.

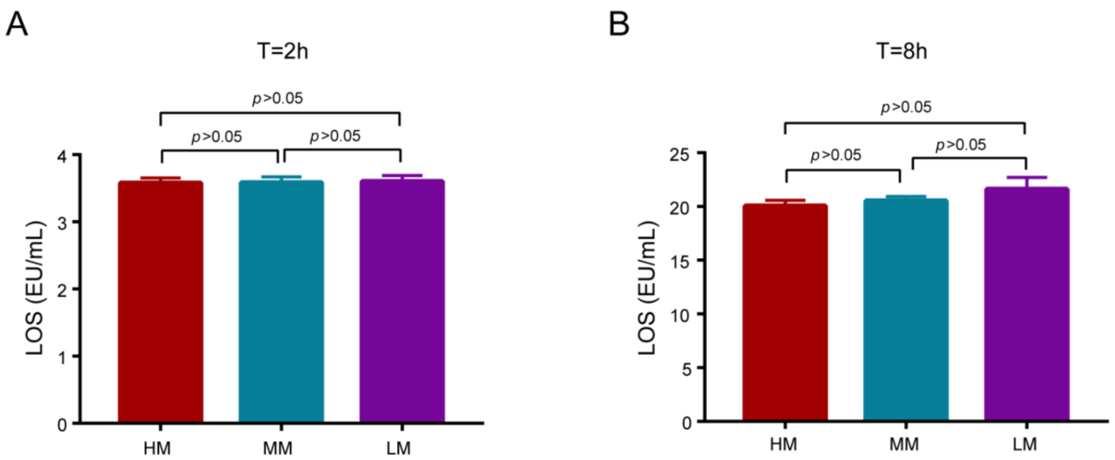

Figure S6 No differences of LOS content are detected in the supernatant after infected with mucoid *A. baumannii*. (A) Determination of LOS content in the supernatant of cell cultures in the absence of intracellular bacteria. MH-S cells are infected with mucoid *A. baumannii* strains (MOI=10) for 2 h. Then the content of LOS in supernatant was determined by Limulus Amoebocyte Lysate test kit. (B) Determination of LOS content in the supernatant of cell cultures with intracellular bacteria observed. All experiments were performed as three biologically independent experiments, and the mean ± s.d. was shown. *P* values were determined using an unpaired, two-tailed Student's *t*-test.

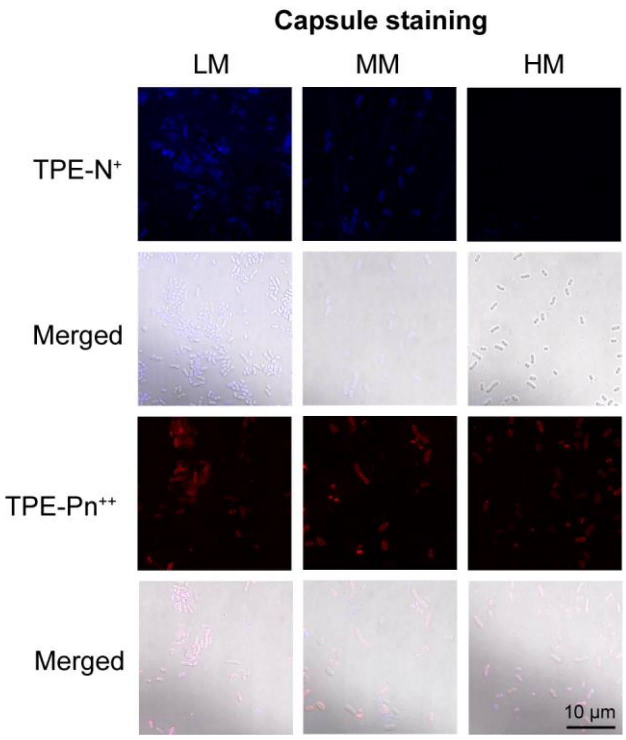

Figure S7 Confocal images of 20 μmol/L single charged probes TPE-N^{+} and double charged probes TPE-Pn^{++}. Scale bar = 10 μm.

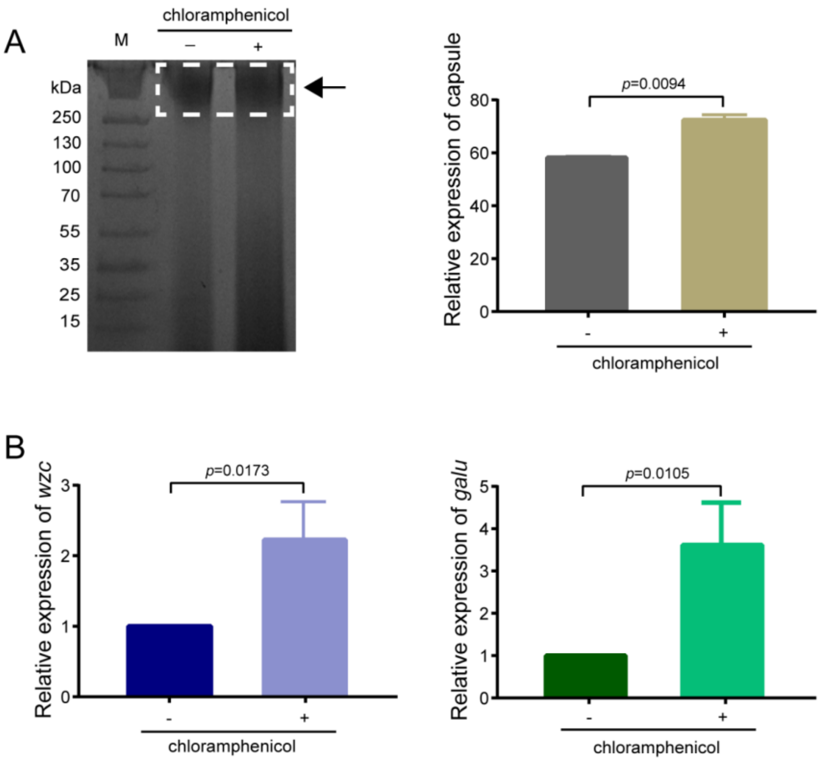

Figure S8 Chloramphenicol induces the increase of capsular production. (A) Image of capsule difference of the LM, MM, and HM strains separated by SDS-PAGE and stained with alcian blue (left). Quantitative analysis of the capsular abundance in the LM, MM, and HM strains (right). (B) The mRNA expression of *galU* and *wzc* in ATCC 17978 strains after 2 h treated with chloramphenicol (10 μg/mL). All experiments were performed as three biologically independent experiments, and the mean ± s.d. was shown. *P* values were determined using an unpaired, two-tailed Student’s *t*-test.

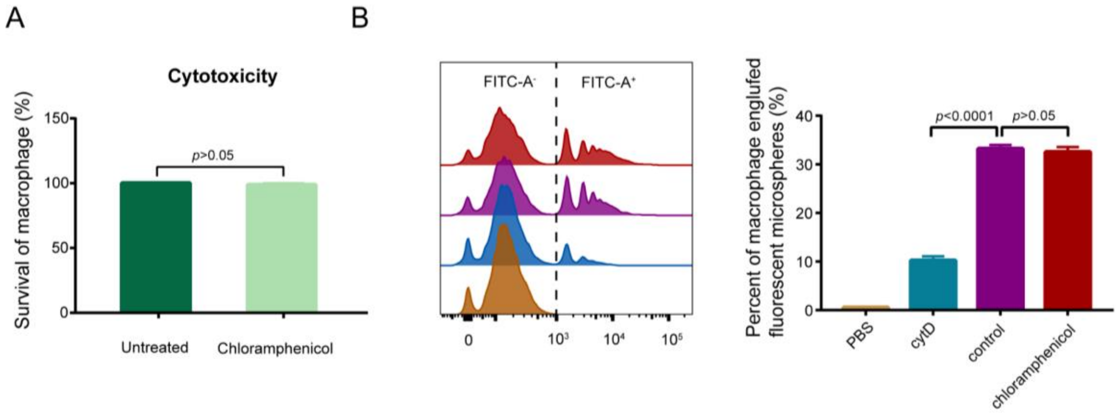

Figure S9 Chloramphenicol shows no cytotoxicity to MH-S cells and has no influence on the phagocytic activity to MH-S cells. (A) Cytotoxicity of chloramphenicol to MH-S cells. MH-S cells are treated with chloramphenicol (10 μg/mL) for 4 h. (B) Fluorescent microsphere uptake assay of macrophages after incubated with chloramphenicol. Representative histograms of fluorescent microspheres engulfed by macrophages (left). Quantitative of microspheres-positive cells. 10,000 events collected per condition for flow cytometry, gated for singlets via FSC/SSC, fluorescence gate set to exclude 99% of isotype control and copied across samples ran in parallel. All experiments were performed as three biologically independent experiments, and the mean ± s.d. was shown. *P* values were determined using an unpaired, two-tailed Student's *t*-test.

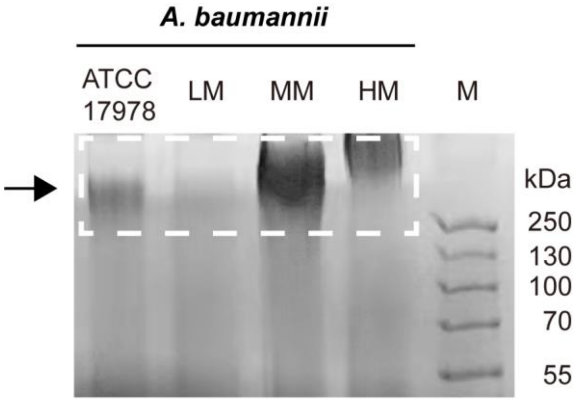

Figure S10 Capsular polysaccharide are different in LM, MM, and HM strains. ATCC 17978 is used as the basic control, capsules are extracted and analyzed by SDS-PAGE and stained with alcian blue. Black arrow indicates capsule. M, marker.

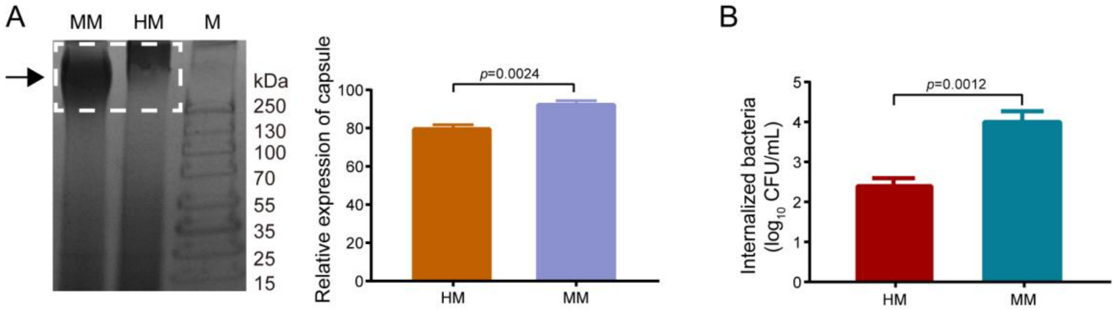

Figure S11 High MW capsule enhances anti-phagocytic activity to host cells. (A) Capsule are stained by alcian blue (left). The black arrow indicates the capsular polysaccharide. M, marker. Quantitative of the capsular polysaccharide in the HM and MM strains (right). (B) Intracellular bacteria quantification of the MH-S cells. MH-S cells are infected with *A. baumannii* strains (MOI=10) for 8 h. All experiments were performed as three biologically independent experiments, and the mean ± s.d. was shown. *P* values were determined using an unpaired, two-tailed Student's *t*-test.
